# Description and molecular phylogeny of *Mesocriconema abolafiai* n. sp. (Nematoda: Criconematidae) from Iran

**DOI:** 10.21307/jofnem-2020-048

**Published:** 2020-05-25

**Authors:** Hossein Mirbabaei Karani, Ali Eskandari, Reza Ghaderi, Akbar Karegar

**Affiliations:** 1 Department of Plant Protection, Faculty of Agriculture, University of Zanjan, 45371-38791, Zanjan, Iran; 2 Department of Plant Protection, School of Agriculture, Shiraz University, 71441-65186, Shiraz, Iran

**Keywords:** Criconematidae, *Mesocriconema*, *M. abolafiai* n. sp., Phylogeny, Morphology, Morphometric, New species, 28S rRNA, 18S rRNA, ITS rRNA

## Abstract

*Mesocriconema abolafiai* n. sp. is described by morphological, morphometric, and molecular approaches. The new species is characterized by a body slightly curved with 402 to 612 μm length, 90 to 113 cuticular body annuli with smooth to irregular margins lacking of crenation with not more than one anastomoses, lip region not offset, small flattened submedian lobes, stylet robust (52.8-60.0 μm) with well-developed knobs, open vulva with simple anterior lip, straight vagina, filled spermatheca with globular sperms, presence of males, and conical-acute tail with last annulus bilobed or rounded. Discussions are made on the characterization of *M. abolafiai* n. sp. from the most closely related species, *M. ozarkiense*, and several other species having similar tail shape. Furthermore, results of phylogenetic analyses inferred from D2 to D3 expansion fragments of 28S rRNA, 18S rRNA, and ITS rRNA gene sequences revealed the phylogenetic position of the new species within representatives of Criconematidae and supported morphological justifications for considering this population from Iran as a new species in the genus *Mesocriconema*.

Ring nematodes of the genus *Mesocriconema* (Andrássy, 1965) are damaging root ectoparasites of many economical important crops (Cordero et al., 2012). This genus was first proposed for species of the genus *Criconemoides* (Taylor, 1936) *sensu lato* with crenated margins of annuli (Andrássy, 1965). Simultaneously and independently, De Grisse and Loof (1965) proposed to divide the large genus *Criconemoides* into several genera including *Macroposthonia* with type species *M. annulata* (De Man, 1880) being among them (De Grisse and Loof, 1965). Luc and Raski (1981) declared *Criconemoides* and *Macroposthonia* as *genera dubia* and placed most of their species in the genus *Criconemella* (De Grisse and Loof, 1965). Based upon SEM microscopy and discussing on plesiomorphic and apomorphic states of characters, Loof and De Grisse (1989) replaced the generic name *Macroposthonia* by the oldest available synonym *Mesocriconema* and revalidated *Criconemoides* based on the arguments of Loof and De Grisse (1967), but Siddiqi (2000) still considered *Macroposthonia* as a valid name. Brzeski et al. (2002) accepted this synonymy and provided a compendium of the genus *Mesocriconema* with 90 species (species having open vulva and submedian lobes arising from reduced pseudolips). Moreover, they considered that *Mesocriconema* differs from *Criconemoides* (species with closed vulva and pseudolips not reduced). Geraert (2010) replaced some species in the genus *Neobakernema* (Ebsary, 1981b) by validation of this genus and listed 90 valid species under *Mesocriconema* excluding *M. lamothei* from Mexico (Cid del Prado Vera, 2009) that was not included in the list. After that, three other species have been identified. *Mesocriconema ozarkiense* (Cordero et al., 2012) was described from Ozark National Forest in Washington, USA (Cordero et al., 2012). *Mesocriconema ericaceum* (Powers et al., 2016) was differentiated from *M. xenoplax* (Raski, 1952; Loof and De Grisse, 1989) by morphological characters and mitochondrial genome (COI) analysis (Powers et al., 2016). *Mesocriconema nebraskense* (Olson et al., 2017) was described as a monosexual, cryptic species sympatrically distributed with its cryptic counterpart, *M. curvatum* (Raski, 1952; Loof and De Grisse, 1989; Olson et al., 2017). In this paper, we describe the new species *M. abolafiai* n. sp., based on morphological and molecular characteristics.

## Material and methods

### Nematode populations and morphological characterization

The specimens were recovered from two localities in Dehdasht and Basht (Kohgiluyeh and Boyer-Ahmad province, Southern Iran). The nematodes were extracted from the soil around roots of a grass (*Phragmites* sp.) using the combination of sieving and centrifugal-flotation method (Jenkins, 1964), killed and fixed by hot FPG (4:1:1, formaldehyde: propionic acid: glycerin), processed to anhydrous glycerin (De Grisse, 1969), and finally mounted in glycerin on permanent slides using paraffin wax. Specimens preserved in glycerin were selected for observation under SEM according to Abolafia (2015). The nematodes were hydrated in distilled water, dehydrated in a graded ethanol-acetone series, critical point dried, coated with gold, and observed with a Zeiss Merlin microscope (5 kV) (Zeiss, Oberkochen, Germany).

Morphometric and morphological characters of the nematode populations were studied by a light microscope, equipped with a Dino-eye microscope eyepiece camera in conjunction with its Dino Capture version 2.0 software. The nematode species identified by using data documented by Brzeski et al. (2002) and Geraert (2010), as well as by comparison with recently published descriptions (Cid del Prado Vera, 2009; Cordero et al., 2012; Powers et al., 2016; Olson et al., 2017).

### DNA extraction

For molecular analysis, DNA was extracted from a single specimen, and three amplifications were conducted on that single specimen. A single female nematode was transferred into a drop of distilled water on a microscopic slide and examined under a light microscope. The nematode specimen was transferred into deionized water, washed three times and then put into an Eppendorf tube with 8 μl distilled water. Then, 12 μl lysis buffer (500 mM KCl, 100 mM Tris-HCL pH 8, 15 mM MgCl_2_, 10 mM DTT, 4.5% Tween 20) and 2 μl proteinase K were added to the Eppendorf tube. Nematode specimen was crushed with a microhomogenizer during 2 min. The tubes were incubated at 65°C for an hour and then at 95°C for 15 min (Tanha Maafi et al., 2003).

### PCR amplification and sequencing

For DNA amplification the protocol described by Tanha Maafi et al. (2003) was used. The D2 to D3 expansion regions of the 28S rRNA gene was amplified with the forward D2A (5´-ACAAGTACCGTGAGGGAAAGTTG-3´) and the reverse D3B (5´-TCGGAAGGAACCAGCTACTA-3´) primers (Nunn, 1992). The 18S rRNA was amplified as two partially overlapping fragments, using three universal and one nematode-specific primer (1912R). First 18S fragment forward primer 988F (5´-CTCAAAGATTAAGCCATGC-3´) and reverse primer 1912R (5´-TTTACGGTCAGAACTAGGG-3´) and the second fragment forward primer 1813F (5´-CTGCGTGAGAGGTGAAAT-3´) and reverse 2646R (5´-GCTACCTTGTTACGACTTTT-3´) were used in the PCR reactions for the amplification of the 18S rRNA gene (Holterman et al., 2006). The ITS1-5.8S-ITS2 regions were amplified with the forward TW81 (5´-GTTTCCGTAGGTGAACCTGC-3´) and reverse AB28 (5´-ATATGCTTAAGTTCAGCGGGT-3´) primers (Joyce et al., 1994).

The PCR products were purified using the QIAquick Gel Extraction Kit (Takapozist, Iran) according to the manufacturer’s instruction and used for direct sequencing. The PCR products were sequenced in both directions (BioNeer Inc., Korea). The newly obtained sequences of the new species were submitted to GenBank database under accession numbers MN334221 for the 18S, MN334222 for the 28S D2-D3, and MN334228 for the ITS sequences.

### Phylogenetic analysis

The sequences of the studied specimens were compared with sequences of other taxa in GenBank, and then, the closest sequences were selected for phylogenetic analyses. The sequences of 18S rRNA and D2 to D3 segments of 28S rRNA were aligned with ClustalX 1.83 (Thompson et al., 1997), using default parameter values and were manually edited if necessary. The best fitted model of DNA evolution was obtained using jModelTest v. 2 (Darriba et al., 2012) with the Akaike information criterion (AIC). The best-fit nucleotide substitution models were considered to be GTR + I + G for 18S and 28S, and SYM + G for ITS. The phylogenetic tree of sequences was inferred by the Bayesian method using MrBayes 3.1.2 (Ronquist and Huelsenbeck, 2003). Four MCMC chains for 1,000,000 generations were run. The Markov chains were sampled at intervals of 100 generations. Two runs were conducted for analysis. After discarding burn-in samples and evaluating convergence, the remaining samples were retained for further analyses. The topologies were used to generate a 50% majority rule consensus tree. Posterior probabilities (PP) are given for appropriate clades. Pairwise divergences between taxa were computed as absolute distance values and as percentage mean distance values based on whole alignment, with adjustment for missing data with PAUP* 4.0b 10 (Swofford, 2002). Trees were visualized using TreeView (Page, 1996).

## Results

### Systematics

#### *Mesocriconema abolafiai* n. sp.

**Figure 1:**
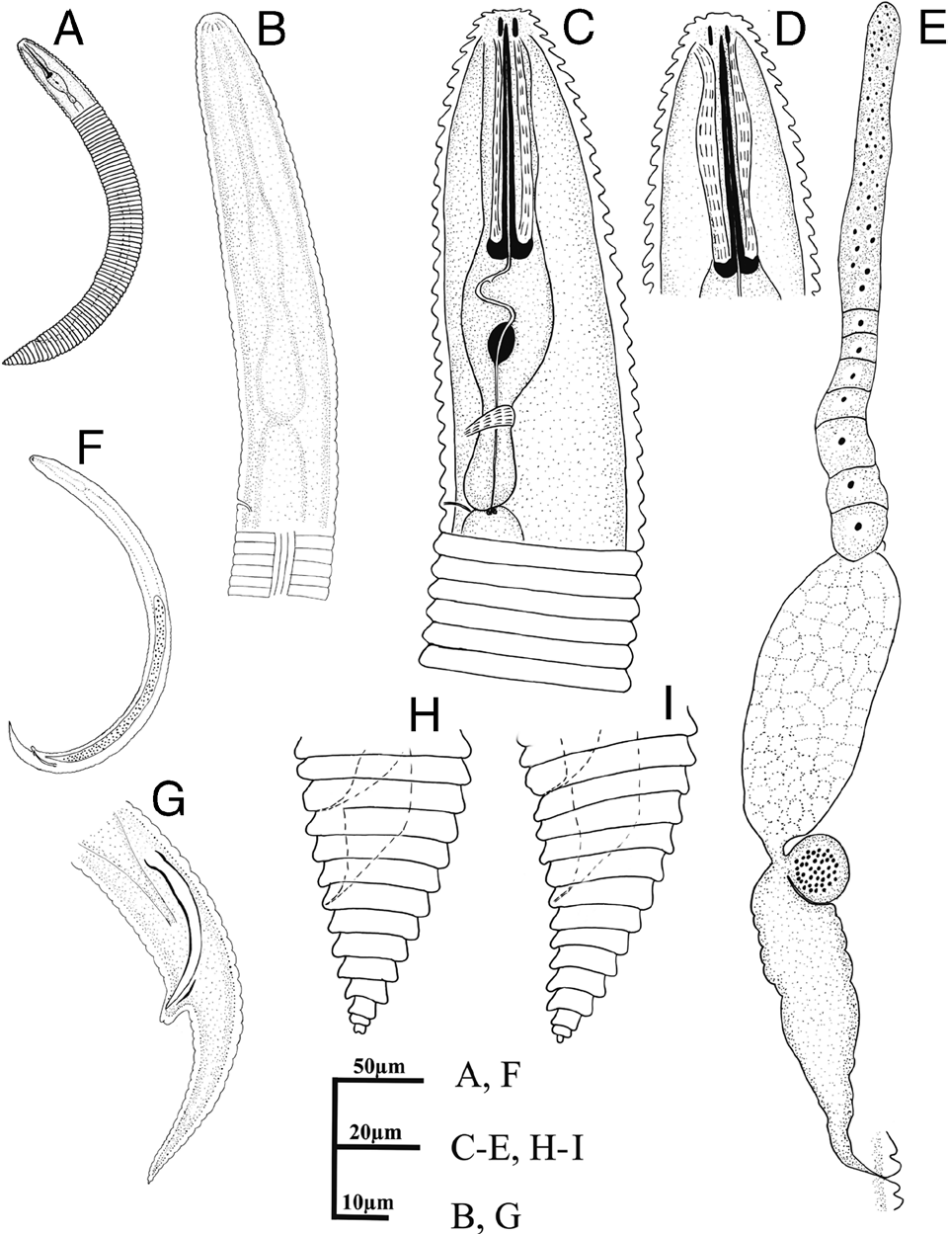
Diagnostic drawings of *Mesocriconema abolafiai* n. sp. Female (A, C-E, H, I) and Male (B, F, G). A, F: entire body; B-D: anterior end and pharyngeal region; E: reproductive system; G-I: posterior end.

**Figure 2:**
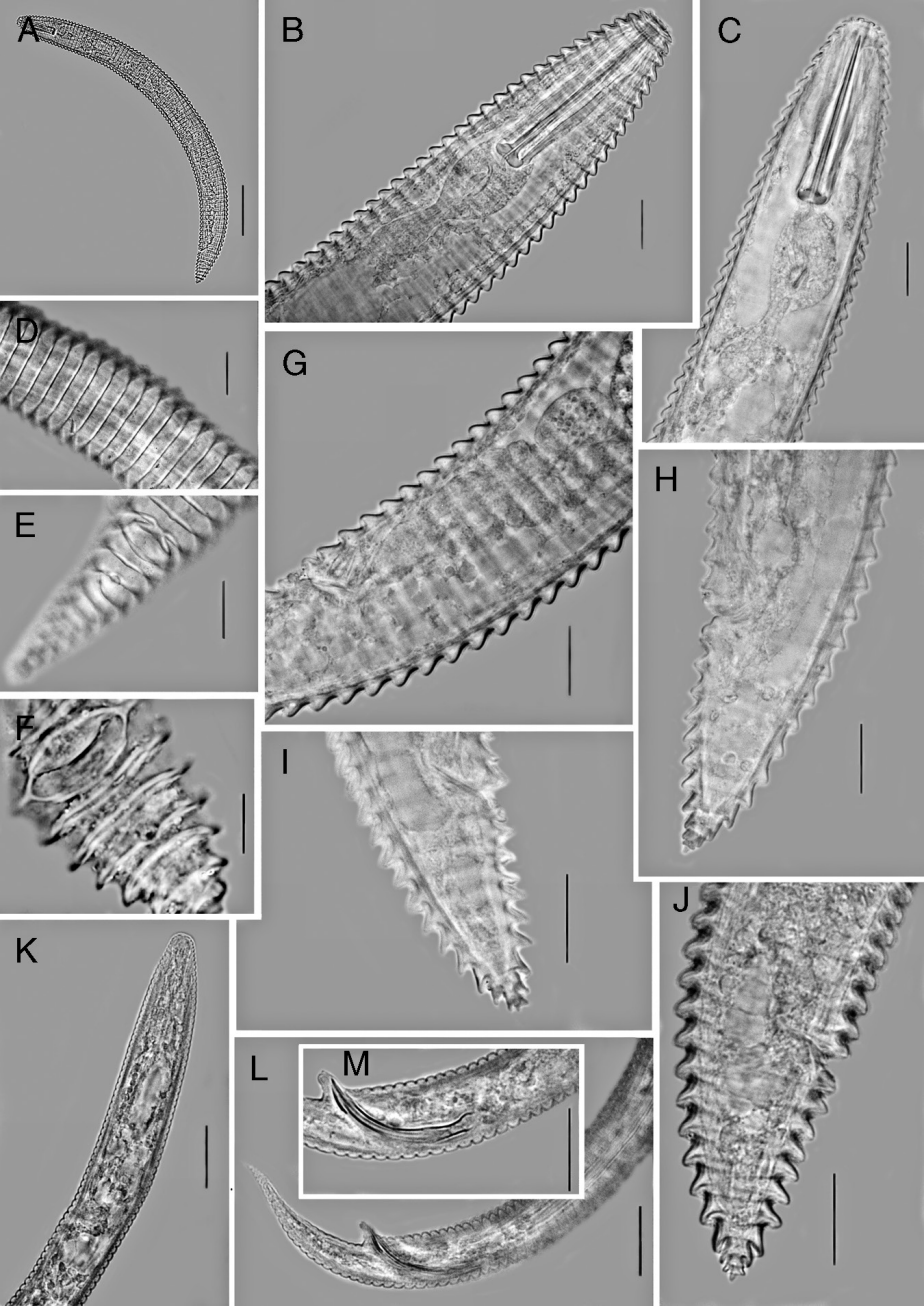
Light micrographs of *Mesocriconema abolafiai* n. sp. Female (A-J) and male (K-M). A: entire body; B, C and K: anterior end and pharyngeal region; D: cuticle at mid-body; E and F: cuticle at posterior end; G: vulval region and part of reproductive system; H-J and L: posterior end; M: spicule, gubernaculum and cloaca. (Scale bars: A = 50 μm; B-M = 10 μm).

**Figure 3:**
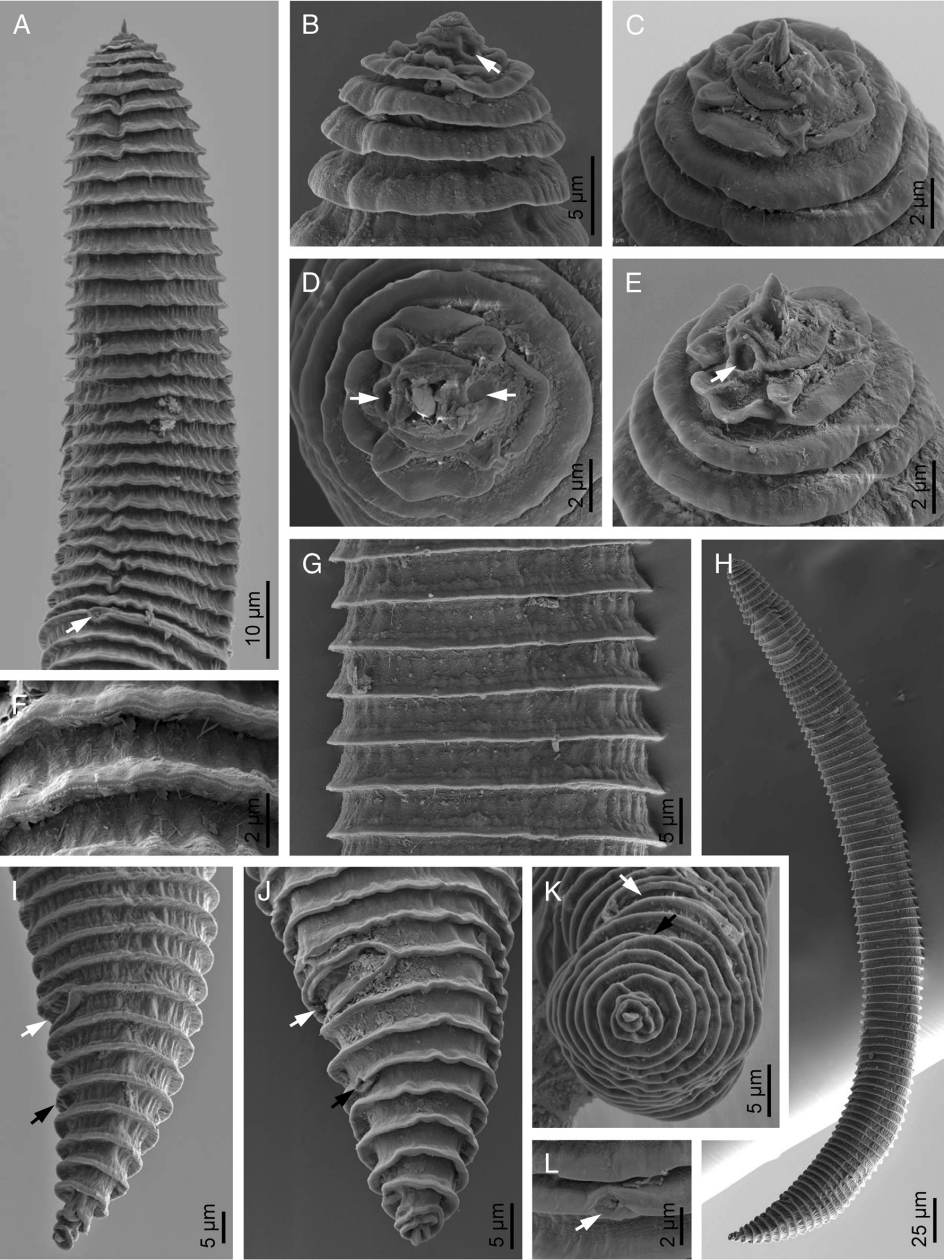
SEM micrograghs of *Mesocriconema abolafiai* n. sp. Female (A-L). A: anterior end (arrow pointing the secretory–excretory pore); B-E: lip region in sublateral, left subventral, frontal and right subventral views, respectively (arrows pointing the amphids); F: annuli; G: cuticle at mid-body; H: entire body; I-K: posterior end in lateral, subventral and terminal views, respectively (white arrow pointing the vulva, black arrow pointing the anus); L: excretory pore (arrow).

(Figs 1-3; Table 1).

**Table 1.** Morphometric characters of *Mesocriconema abolafiai* n. sp. (measurements are in μm and in the form of average ± s.d. (range)).

		Dehdasht population		Basht population
Characters	Holotype	10 paratype females	3 paratype males	5 females
L	540	540 ± 65 (402–612)	471 ± 33.6 (435–502)	519 ± 65 (425–605)
a	14.9	14 ± 1.5 (10.3–15.3)	24.6 ± 3.2 (20.9–27.1)	12.6 ± 1.2 (10.9–14.4)
b	4.8	4.8 ± 0.6 (3.2–5.7)	24.6	4.3 ± 0.5 (3.5–5.0)
c	14.5	15.8 ± 2.4 (12.1–19.5)	10.5 ± 0.5 (9.9–11)	19.1 ± 2.4 (16.3–21.9)
c´	1.4	1.3 ± 0 (1.3–1.4)	3.3 ± 0.5 (2.9–3.9)	1.3 ± 0 (1.1–1.4)
V	90.5	90.3 ± 1.1 (87.8–91.9)	–	90.1 ± 0.4 (89.8–90.8)
Stylet	56.9	55.2 ± 1.3 (52.8–57.2)	–	56.8 ± 2.3 (54–60)
Conus	42.3	44 ± 6.3 (38.6–55.0)	–	41.9 ± 1.6 (40.0–44.5)
m (conus/stylet %)	74.4	80.1 ± 11.2 (73.1–100.0)	–	73.7 ± 1.7 (71.9–76.3)
Pharynx	110.7	113 ± 8 (103–124)	59.3	119 ± 8 (105–126)
Post-vulval body length (VL)	50.7	51.8 ± 4.9 (44.6–58.9)	–	51.2 ± 6.7 (43–60)
Secretory-excretory pore	109	108 ± 7 (99–118)	106 ± 12 (92–113)	108 ± 8 (97–118)
Lip region-vulva	485.2	488 ± 62 (353–553)		468 ± 58.6 (382–545)
Lip region-anus	498.9	506 ± 64.1 (372–575)	426 ± 29.5 (396–455)	492 ± 63.8 (399–577)
Vulva-anus	24.0	22 ± 2.9 (17.1–26.0)	–	24 ± 5.3 (17–32)
Tail length	37.0	34.3 ± 3.6 (28.0–39.3)	44.8 ± 4.5 (39.5–47.5)	27.2 ± 2.9 (23–31)
Body width	36.1	38.3 ± 2.4 (34.1–42.0)	19.2 ± 1.6 (17.5–20.7)	41.2 ± 4.3 (34–46)
Vulval body width (VB)	30.4	30.1 ± 1.6 (27.2–32.3)	–	30.7 ± 1.9 (27.5–32.5)
VL/VB	1.7	1.7 ± 0.1 (1.6–1.8)	–	1.6 ± 0.1 (1.5–1.8)
Annulus width	5.2	5.4 ± 0.7 (4.1–6.5)	2.8 ± 0.4 (2.3–3.2)	5.7 ± 0.6 (4.9–6.8)
R	104.0	104.2 ± 4 (97–113)	132	97 ± 4.3 (90–101)
Rst	16.0	15.5 ± 0.8 (14–17)	–	14.5 ± 0.5 (14–15)
Rph	26.0	25 ± 1.4 (23–28)	–	24.8 ± 1.9 (23–28)
Rexp	29	23.2 ± 4.2 (19–30)	49.6 ± 2 (48–52)	27.1 ± 4.2 (24–29)
RV	12.0	12 ± 0.8 (11–14)	–	12.8 ± 0.8 (12–14)
Ran	8.0	9 ± 0.9 (8–10)	–	7.4 ± 0.5 (7–8)
RVan	3.0	3.6 ± 0.4 (3–4)	–	3.6 ± 0.8 (3–5)
St/L × 100	10.6	10.3 ± 1.3 (9.2–13.6)	–	11 ± 1.1 (9.4–12.7)
Spicules	–	–	34.2 ± 0.7 (33.6–35.0)	–
Gubernaculum	–	–	6.2 ± 0.9 (5.3–7.2)	–

### Description

#### Female

Body is slightly curved ventrally when relaxed by heat, assuming an open C-shaped. Cuticular annuli are retrorse, and margins are smooth to irregular across the entire body, without any hint of crenation. Not more than one anastomosis is observed. Cephalic region is not offset, tapering and slightly conical. Oral disc has rounded edges, and slit-like amphidial apertures are located laterally on the disc. Four small flattened submedian lobes are visible at the same level with a labial plate. Submedian lobes in SEM resemble a tongue with a central, longitudinal crease, not fused with labial plates. First body annulus is slightly smaller than the second annulus, not retrorse. Stylet is robust, with well-developed knobs that possess moderate anterior projections and 9 to 11 μm in width. Secretory–excretory pore is located at 27 to 30 annuli from the anterior end, almost at 4 to 5 annuli behind the pharyngeal basal bulb, which is small, pyriform, 10 to 13.5 μm in width and 18 to 20 μm in length. Female genital gonad is outstretched and spermatheca is slightly oval, offset from gonad, filled with globular sperm cells about 1 μm in diameter. Vagina is straight, occasionally slightly curved. Vulva is open with simple anterior lip. The post-vulval region of the body tapers gradually, ending in a pointed terminus or a small bilobed end annulus. SEM reveals that the anal opening is located usually three annuli posterior to the vulva.

#### Male

Body is vermiform and curved ventrally when fixed by heat treatment. Lateral field has four distinct longitudinal incisures. Lip region has distinct transverse striation, 8 to 9 μm wide and 5 to 7 μm high. Stylet and pharynx are degenerated. Spicules (33.6-35.0 μm in length) are slender and tylenchoid, ending to a distinct penial tube (4-5 μm in length). Gubernaculum is simple and slightly curved. Bursa not is observed. Tail is elongated-conical, ending to a pointed terminus.

#### Juvenile

Similar to female in general characteristics. Body is straight or slightly curved ventrally after fixation. Annuli are retrorse, lacking any crenation and ornamentation. Total number of annuli approximately equals to that of females, but annuli are narrower than of females (average 4.4 vs 5.5 μm).

#### Diagnosis

*Mesocriconema abolafiai* n. sp. is characterized by 90 to 113 cuticular annuli with smooth to irregular margins lacking crenation, small and flattened submedian lobes, stylet 52.8 to 60.0 μm long, open vulva with a simple anterior vulval lip, straight vagina, spermatheca filled with globular sperm, presence of males, and conical-acute tail with last annulus bilobed or rounded.

#### Relationships

*Mesocriconema abolafiai* n. sp. is characterized by having flattened submedian lobes groups with *M. antipolitanum* (De Guiran, 1963); *M. citricola* (Siddiqi, 1965; Loof and De Grisse, 1989); *M. juliae* (Crozzoli and Lamberti, 2001); *M. napoense* (Talavera and Hunt, 1997); *M. oostenbrinki* (Loof, 1964); *M. ozarkiense* (Cordero et al., 2012); *M. paralineolatum* (Rashid et al., 1987); *M. planilobatum* (Ta1avera and Hunt, 1997); and *M. rusticum* (Khan et al., 1976) in the diagnostic compendium developed by Brzeski et al. (2002).

*Mesocriconema abolafiai* n. sp. can be distinguished from *M. antipolitanum* and *M. rusticum* by differences in the size of submedian lobes (small vs large), tail shape (conical vs rounded), spermatheca (filled vs empty) and occurring of males (present vs absent). It differs from *M. citricola* by a different shape of the anterior vulval lip (simple vs bilobed) and higher number of cuticular annuli (90-113 vs 73-78). It can be differentiated from *M. juliae* by stylet length (52.8-60.0 vs 79-86 μm) and shape of the anterior vulval lip (simple vs bilobed). *Mesocriconema abolafiai* n. sp. can be distinguished from *M. napoense*, *M. paralineolatum*, and *M. planilobatum* by the number of cuticular annuli (90-113 vs 73-79, 82-88, 75-84, respectively) and vagina direction (straight vs sigmoid). It differs from *M. oostenbrinki* by a different shape of the anterior vulval lip (simple vs bilobed), the number of cuticular annuli (90-113 vs 84-94), and vagina direction (straight vs sigmoid). Our new species can be distinguished from *M. ozarkiense* by differences in the posterior end of cuticular annuli on post-vulval region (smooth vs crenated), spermatheca (filled vs empty), occurring of males (presence vs absence), vagina direction (straight vs sigmoid), and VL/VB ratio (1.5-1.9 vs 1.0-1.4).

Regarding general morphometric characters and tail shape, our populations can also come similar to *M. denoudeni* (De Grisse, 1967; Loof and De Grisse, 1989); *M. jessiense* (Van den Berg, 1992, 1994); *M. reedi* (Diab and Jenkins, 1966; Loof and De Grisse, 1989); *M. raskiense* (De Grisse, 1964; Andrássy, 1965); *M. vadense* (Loof, 1964; Loof and De Grisse, 1989); *M. kirjanovae* (Andrássy, 1962; Loof and De Grisse, 1989); *M. paradenoudeni* (Rashid et al., 1987; Loof and De Grisse, 1989); and *M. parareedi* (Ebsary, 1981a; Loof and De Grisse, 1989). However, our populations can be differentiated from *M. denoudeni* by a different tail terminus shape (conical-acute vs conical-rounded), the number of post-vulval annuli (11-14 vs 8-11), VL/VB ratio (1.5-1.9 vs 1.0-1.3), and presence of males. The new species differs from *M. jessiense* and *M. reedi* by having more annuli at post-vulval region (11-14 vs 8-9 and 9-10) and higher value for the VL/VB ratio (1.5-1.9 vs 0.8-1.1 and 1.1-1.3), and differs from *M. reedi* by having a larger body size (402-612 vs 360-470 μm). In comparison with *M. raskiense*, it has more annuli throughout body (90-113 vs 62-72), and a different structure of cuticular annuli (smooth and without anastomoses vs crenated with anastomoses at mid-body). It also differs from *M. vadense* by the number of cuticular annuli (90-113 vs 70-81), the number of post-vulval annuli (11-14 vs 7-10), VL/VB ratio (1.5-1.9 vs 0.8-1.3), and tail shape (conical-acute vs conical-rounded).

*M. abolafiai* n. sp. can be further distinguished from *M. kirjanovae*, *M. citricola*, *M. paradenoudeni*, and *M. parareedi* by a different shape of the anterior vulval lip (simple vs bilobed), and variations in the number of cuticular annuli (90-113 vs 79-89, 73-78, 102-130, and 111-121, respectively).

The males recovered in the type population have a unique elongated tail with pointed terminus, which only could be observed in *M. raskiense* and *M. vadense*. Spicules in our population are comparable with those of *M. vadense* (33-35 vs 30-34 μm) but shorter than those in *M. raskiense* (33.6-35.0 vs 38-43 μm). Males in some other species including *M. brevicauda* (Van den Berg and Spaull, 1985; Loof and De Grisse, 1989); *M. curvatum*, *M. involutum* (Loof, 1987, 1989); *M. irregulare* (De Grisse, 1964; Loof and De Grisse, 1989); *M. juliae* and *M. oostenbrinki* have more or less similar tails but shorter in size or with a different terminus shape.

#### Type host and locality

The type population was found from a canebrake in Dehdasht, Kohgiloyeh and Boyer-Ahmad province (30°49.42´N, 51°28.91´E). The other population was recovered from the rhizosphere of dog-rose shrubs (*Rosa canina* L.) in Basht, Kohgiloyeh and Boyer-Ahmad province (30°19.29´N, 51°15.04´E) during April 2017 by the first author.

#### Type specimens

Holotype, 10 paratype females and three paratype males, as well as five female specimens from the other recovered population were deposited in the nematode collection of the Department of Plant Protection, College of Agriculture, University of Zanjan, Zanjan, Iran.

#### Etymology

The species epithet refers to the name of Dr. Joaquín Abolafia, the well-known nematologist from University of Jaén, Spain, who works on nematode systematics.

#### Phylogenetic relationships

**Figure 4:**
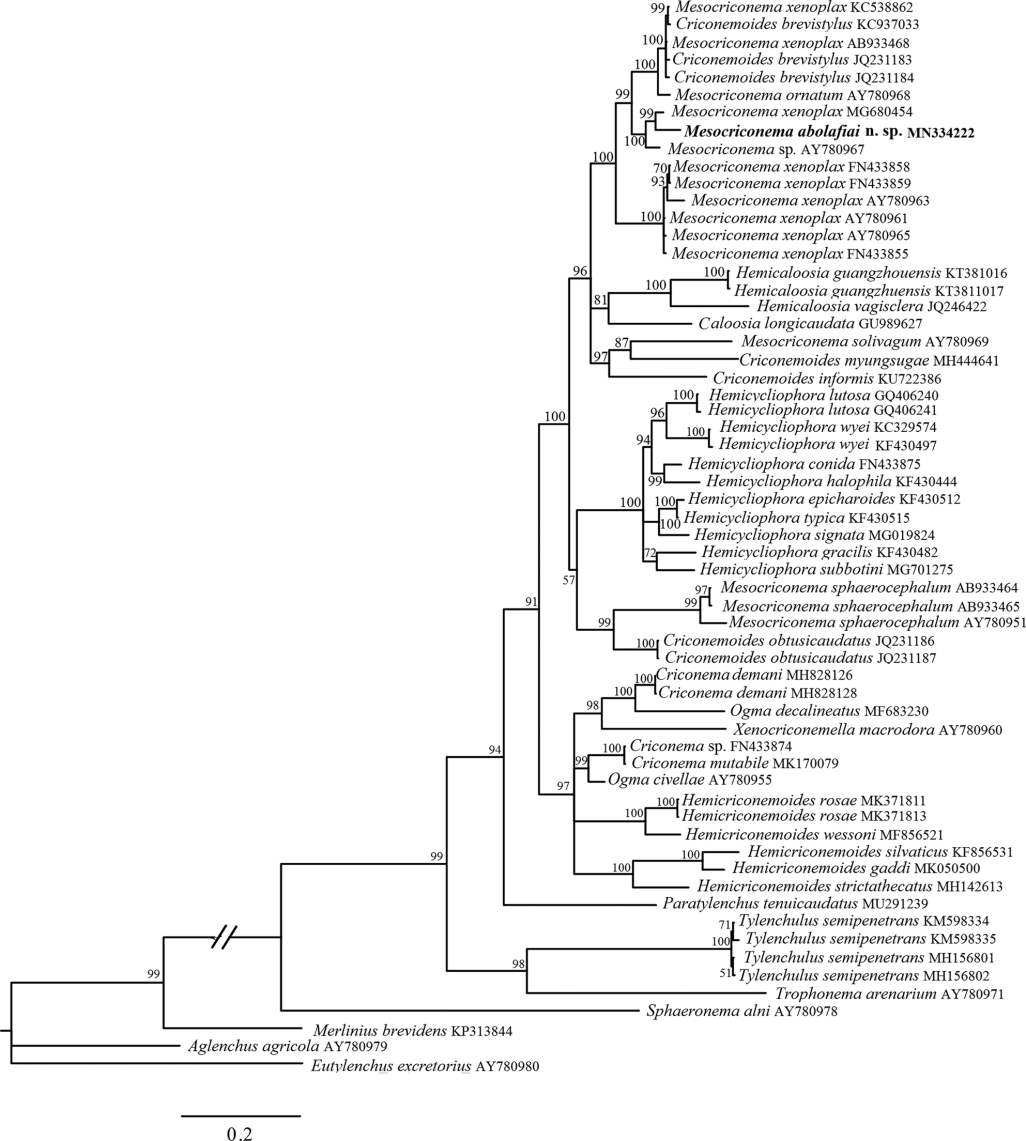
Bayesian 50% majority rule consensus tree as inferred from the D2 to D3 expansion segments of 28S rRNA gene dataset of Criconematoidea under the general time reversible model of sequence evolution with correction for invariable sites and a gamma-shaped distribution (GTR + I + G). Posterior probabilities more than 50% are given for appropriate clades. The new obtained sequence in this study is indicated in bold. Scale bar=expected changes per site.

The 28S alignment was 738 bp long and consisted of 58 sequences as ingroups and three sequences, including *Aglenchus agricola* (Andrássy, 1954; De Man 1884) (AY780979), *Eutylenchus excretorius* (Sher et al., 1966) (AY780980), and *Merlinius brevidens* (Allen, 1955; Siddiqi, 1970) (KP313844), as outgroups (Table 2). Phylogenetic relationships of *M. abolafiai* n. sp. with other representatives of Criconematidae (Taylor, 1936; Thorne, 1949) inferred from the analysis of D2 to D3 expansion fragments of 28S rRNA gene sequences with collapsed branches, with PP less than 50%, are given in Figure 4. In this tree, *M. abolafiai* n. sp. formed a cluster with an isolate of *M. xenoplax* (MG680454) and an unnamed population (AY780967). Partial 28S rRNA sequences of the *M. abolafiai* n. sp. from Iran show about 23 bp (3%) difference with the closet species according to 28S tree (*M. xenoplax*: MG680454), whereas two species distinguished with some characters such as tail (conical vs subcylindrical) and vagina (straight vs sigmoid) shape. There is not any record of partial 28S rRNA sequences of *M. ozarkience* that it is closest species to *M. abolafiai* n. sp. based on morphological characteristics.

**Table 2.** List of species, collection localities and GenBank accession numbers of individual specimens used in this study for phylogenetic analysis based on 28S rRNA gene.

Species name	GeneBank accession no.	Locality	Species name	GeneBank accession no.	Locality
*Aglenchus agricola*	AY780979	Belgium	*Hemicycliophora typica*	KF430515	South Africa
*Caloosia longicaudata*	GU989627	USA	*H. wyei*	KC329574	USA
*Criconema demani*	MH828126	Russia	*H. wyei*	KF430497	USA
*C. demani*	MH828128	Russia	*Merlinius brevidens*	KP313844	Iran
*C. mutabile*	MK170079	South Africa	*Mesocriconema abolafiai* n. sp.	MN334222	Iran
*Criconema* sp.	FN433874	USA	*M. ornatum*	AY780968	Venezuela
*Criconemoides brevistylus*	JQ231183	South Africa	*M. solivagum*	AY780969	Russia
*C. brevistylus*	JQ231184	South Africa	*Mesocriconema* sp.	AY780967	Italy
*C. brevistylus*	KC937033	China	*M. sphaerocephalum*	AB933464	Japan
*C. informis*	KU722386	Iran	*M. sphaerocephalum*	AB933465	Japan
*C. myungsugae*	MH444641	China	*M. sphaerocephalum*	AY780951	Italy
*C. obtusicaudatus*	JQ231186	South Africa	*M. xenoplax*	AB933468	Japan
*C. obtusicaudatus*	JQ231187	South Africa	*M. xenoplax*	AY780961	Germany
*Eutylenchus excretorius*	AY780980	Germany	*M. xenoplax*	AY780963	USA
*Hemicaloosia guangzhouensis*	KT381016	China	*M. xenoplax*	AY780965	Italy
*H. guangzhouensis*	KT381017	China	*M. xenoplax*	FN433855	USA
*H. vagisclera*	JQ246422	USA	*M. xenoplax*	FN433858	USA
*Hemicriconemoides gaddi*	MK050500	China	*M. xenoplax*	FN433859	USA
*H. rosae*	MK371811	India	*M. xenoplax*	KC538862	USA
*H. rosae*	MK371813	India	*M. xenoplax*	MG680454	Portugal
*H. silvaticus*	KF856531	Japan	*Ogma civellae*	AY780955	Venezuela
*H. strictathecatus*	MH142613	China	*O. decalineatus*	MF683230	South Africa
*H. wessoni*	KF856521	USA	*Paratylenchus tenuicaudatus*	KU291239	Iran
*Hemicycliophora conida*	FN433875	Belgium	*Sphaeronema alni*	AY780978	Germany
*H. epicharoides*	KF430512	Italy	*Trophonema arenarium*	AY780971	Italy
*H. gracilis*	KF430482	USA	*Tylenchulus semipenetrans*	KM598334	Iran
*H. halophila*	KF430444	New Zealand	*T. semipenetrans*	KM598335	Iran
*H. lutosa*	GQ406240	South Africa	*T. semipenetrans*	MH156801	China
*H. lutosa*	GQ406241	South Africa	*T. semipenetrans*	MH156802	China
*H. signata*	MG019824	Mozambique	*Xenocriconemella macrodora*	AY780960	Italy
*H. subbotini*	MG701275	China			

**Figure 5:**
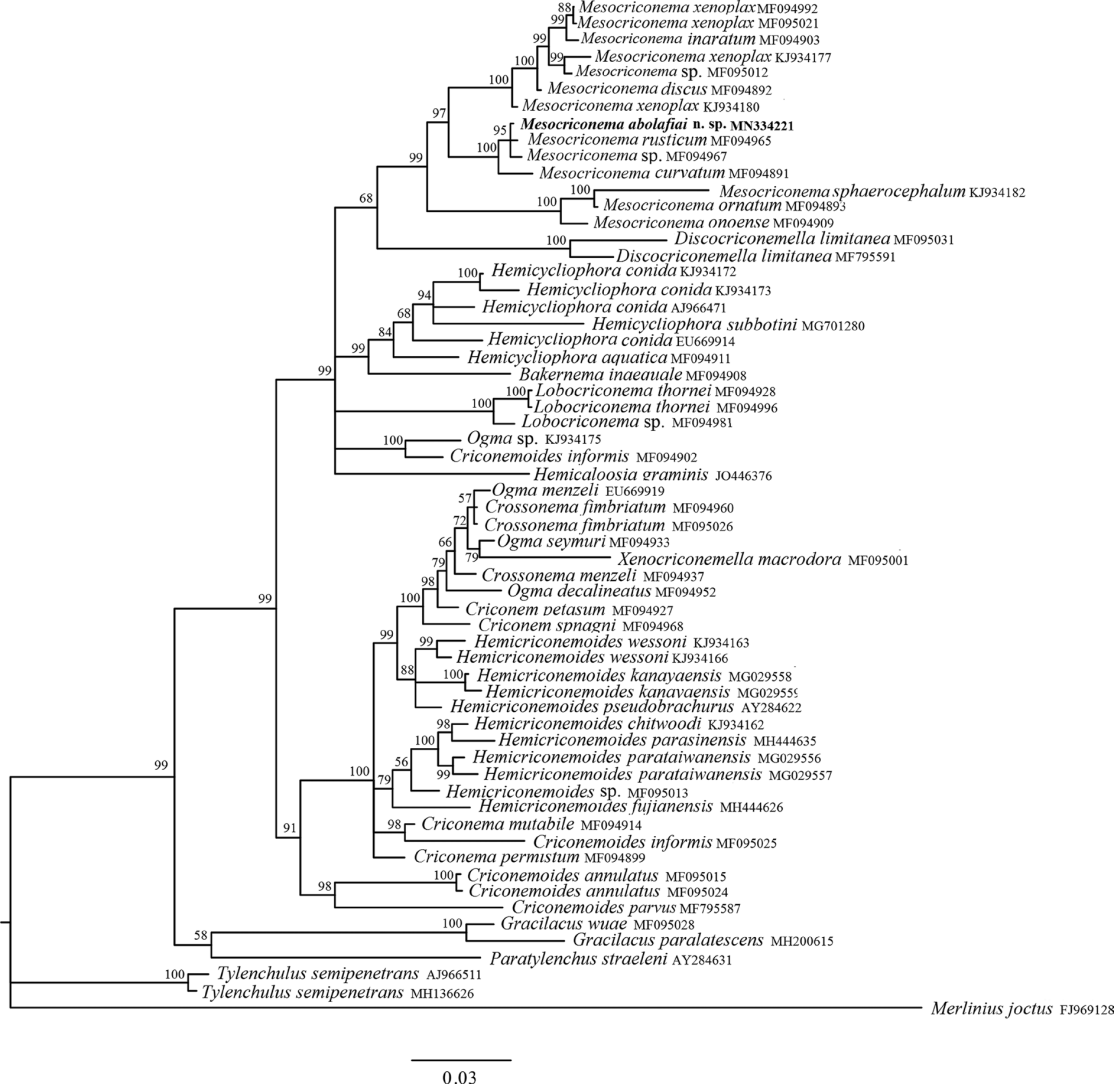
Bayesian 50% majority rule consensus tree as inferred from the 18S rRNA gene dataset of Criconematoidea under the general time reversible model of sequence evolution with correction for invariable sites and a gamma-shaped distribution (GTR + I + G). Posterior probabilities more than 50% are given for appropriate clades. The new obtained sequence in this study is indicated in bold. Scale bar = expected changes per site.

The 18S alignment was 1538 bp long and consisted of 60 sequences as ingroups and *Merlinius joctus* (Thome, 1949; Sher, 1974) as outgroup (Table 3). Phylogenetic relationships of *M. abolafiai* n. sp. with other representatives of Criconematidae inferred from the analysis of 18S rRNA gene sequences with collapsed branches, with PP less than 50%, are given in Figure 5. The new species clustered with *M. rusticum* (MF094965) and *Mesocriconema* sp. (MF094967), all in a sister clade with *M. curvatum* (MF094891). The most important morphologically differences between *M. abolafiai* n. sp. and *M. rusticum* are related to lip region and post-vulval shape, respectively. The lip region in *M. rusticum* is set off with large submedian lobes, anteriorly flattened giving appearance of truncate anterior, but the lip region in *M. abolafiai* n. sp. is not offset, tapering and slightly conical. The post-vulval part is rounded in *M. rusticum* and tail end multi-lobed, mostly bent dorsally, whereas in *M. abolafiai* n. sp. post-vulval region of the body tapers gradually, ending in a pointed terminus or a small bilobed end annulus. The other interesting result inferred from the phylogenetic analysis of the present study is that the 18S rRNA gene is not a good marker for species differentiation in the *Mesocriconema* genus, because there is only one nucleotide difference between *M. abolafiai* n. sp. and *M. rusticum*, whereas these species are well separated based on morphological characteristics.

**Table 3.** List of species, collection localities and GenBank accession numbers of individual specimens used in this study for phylogenetic analysis based on 18S rRNA gene.

Species name	GeneBank accession no.	Locality	Species name	GeneBank accession no.	Locality
*Bakernema inaequale*	MF094908	USA	*H. conida*	AJ966471	GenBank
*Criconema mutabile*	MF094914	USA	*H. conida*	KJ934172	USA
*C. permistum*	MF094899	USA	*H. conida*	KJ934173	USA
*C. petasum*	MF094927	USA	*H. subbotini*	MG701280	China
*C. sphagni*	MF094968	USA	*Lobocriconema* sp.	MF094981	USA
*Criconemoides annulatus*	MF095015	USA	*L. thornei*	MF094928	USA
*C. annulatus*	MF095024	USA	*L. thornei*	MF094996	USA
*C. informis*	MF094902	USA	*Merlinius joctus*	FJ969128	GenBank
*C. informis*	MF095025	USA	*Mesocriconema abolafiai* n. sp.	MN334221	Iran
*C. parvus*	MF795587	China	*M. curvatum*	MF094891	USA
*Crossonema fimbriatum*	MF095026	USA	*M. discus*	MF094892	USA
*C. fimbriatum*	MF094960	USA	*M. inaratum*	MF094903	USA
*C. menzeli*	MF094937	USA	*M. onoense*	MF094909	USA
*Discocriconemella limitanea*	MF795591	China	*M. ornatum*	MF094893	USA
*D. limitanea*	MF095031	Costa Rica	*M. rusticum*	MF094965	USA
*Gracilacus paralatescens*	MH200615	China	*Mesocriconema* sp.	MF094967	USA
*G. wuae*	MF095028	Canada	*Mesocriconema* sp.	MF095012	USA
*Hemicaloosia graminis*	JQ446376	USA	*M. sphaerocephalum*	KJ934182	USA
*Hemicriconemoides. chitwoodi*	KJ934162	USA	*M. xenoplax*	KJ934180	USA
*Hemicriconemoides. fujianensis*	MH444626	China	*M. xenoplax*	KJ934177	USA
*H. kanayaensis*	MG029558	China	*M. xenoplax*	MF095021	USA
*H. kanayaensis*	MG029559	China	*M. xenoplax*	MF094992	USA
*H. parasinensis*	MH444635	China	*Ogma decalineatus*	MF094952	USA
*H. parataiwanensis*	MG029556	China	*O. menzeli*	EU669919	GenBank
*H. parataiwanensis*	MG029557	China	*O. seymouri*	MF094933	USA
*H. pseudobrachyurus*	AY284622	GenBank	*Ogma* sp.	KJ934175	USA
*Hemicriconemoides* sp.	MF095013	Thailand	*Paratylenchus straeleni*	AY284631	GenBank
*H. wessoni*	KJ934163	USA	*Tylenchulus semipenetrans*	MH136626	China
*H. wessoni*	KJ934166	USA	*T. semipenetrans*	AJ966511	UK
*Hemicycliophora aquatica*	MF094911	USA	*Xenocriconemella macrodora*	MF095001	USA
*H. conida*	EU669914	GenBank			

**Figure 6:**
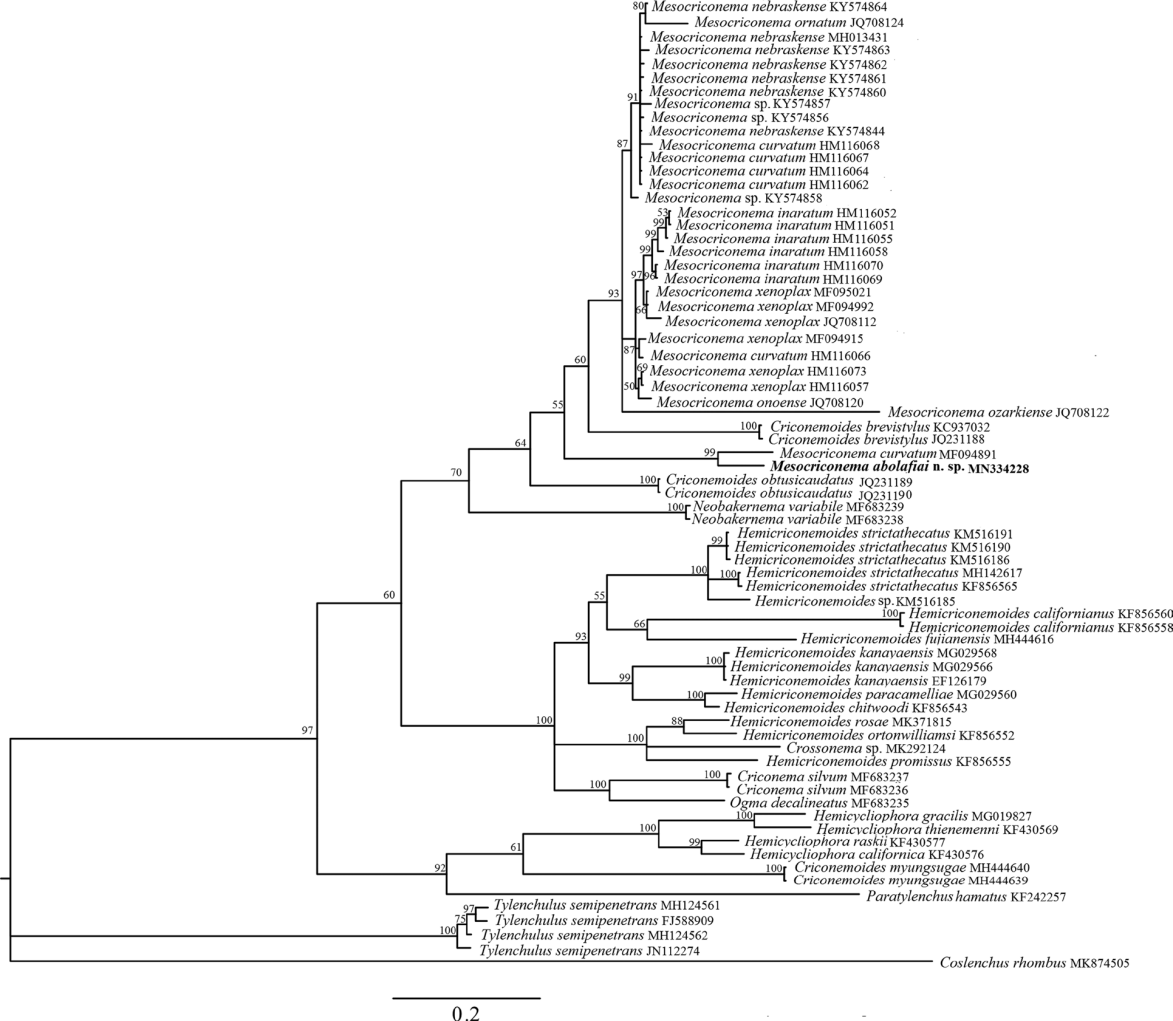
Bayesian 50% majority rule consensus tree as inferred from the ITS rRNA gene dataset of Criconematoidea under the symmetrical model of sequence evolution with a gamma-shaped distribution (SYM + G). Posterior probabilities more than 50% are given for appropriate clades. The new obtained sequence in this study is indicated in bold. Scale bar = expected changes per site.

The ITS alignment was 401 bp long and consisted of 70 sequences as ingroups and *Coslenchus rhombus* (Andrássy, 1982) as outgroup (Table 4). Phylogenetic relationships of *M. abolafiai* n. sp. with other representatives of Criconematidae inferred from the analysis of ITS rRNA gene sequences with collapsed branches, with PP less than 50%, are given in Figure 6. In this tree, the new species clustered with an isolate of *M. curvatum* (MF094891) with 37 bp difference (9.2%), and in a distant position from the morphologically related species, *M. ozarkiense* (JQ708122) with 129 bp difference (32%).

**Table 4.** List of species, collection localities and GenBank accession numbers of individual specimens used in this study for phylogenetic analysis based on ITS rRNA gene.

Species name	GeneBank accession no.	Locality	Species name	GeneBank accession no.	Locality
*Coslenchus rhombus*	MK874505	South Africa	*Mesocriconema curvatum*	MF094891	USA
*Criconema silvum*	MF683236	South Africa	*M. inaratum*	HM116070	USA
*C. silvum*	MF683237	South Africa	*M. inaratum*	HM116069	USA
*Criconemoides brevistylus*	KC937032	China	*M. inaratum*	HM116058	USA
*C. brevistylus*	JQ231188	South Africa	*M. inaratum*	HM116055	USA
*C. myungsugae*	MH444640	China	*M. inaratum*	HM116052	USA
*C. myungsugae*	MH444639	China	*M. inaratum*	HM116051	USA
*C. obtusicaudatus*	JQ231189	South Africa	*M. nebraskense*	MH013431	USA
*C. obtusicaudatus*	JQ231190	South Africa	*M. nebraskense*	KY574844	USA
*Crossonema* sp.	MK292124	USA	*M. nebraskense*	KY574860	USA
*Hemicriconemoides californianus*	KF856558	USA	*M. nebraskense*	KY574861	USA
*H. californianus*	KF856560	USA	*M. nebraskense*	KY574862	USA
*H. chitwoodi*	KF856543	USA	*M. nebraskense*	KY574863	USA
*H. fujianensis*	MH444616	China	*M. nebraskense*	KY574864	USA
*H. kanayaensis*	EF126179	Taiwan	*M. nebraskense*	KY574865	USA
*H. kanayaensis*	MG029566	China	*M. onoense*	JQ708120	USA
*H. kanayaensis*	MG029568	China	*M. ornatum*	JQ708124	USA
*H. ortonwilliamsi*	KF856552	Spain	*M. ozarkiense*	JQ708122	USA
*H. paracamelliae*	MG029560	China	*Mesocriconema* sp.	KY574858	USA
*H. promissus*	KF856555	Spain	*Mesocriconema* sp.	KY574857	USA
*H. rosae*	MK371815	India	*Mesocriconema* sp.	KY574856	USA
*Hemicriconemoides* sp.	KM516185	USA	*M. xenoplax*	JQ708112	USA
*H. strictathecatus*	KF856565	South Africa	*M. xenoplax*	HM116073	USA
*H. strictathecatus*	MH142617	China	*M. xenoplax*	HM116057	USA
*H. strictathecatus*	KM516186	USA	*M. xenoplax*	MF095021	USA
*H. strictathecatus*	KM516190	USA	*M. xenoplax*	MF094992	USA
*H. strictathecatus*	KM516191	USA	*M. xenoplax*	MF094915	USA
*Hemicycliophora californica*	KF430576	USA	*M. xenoplax*	MF094916	USA
*H. gracilis*	MG019827	USA	*Neobakernema variabile*	MF683239	USA
*H. raskii*	KF430577	USA	*N. variabile*	MF683238	USA
*H. thienemanni*	KF430569	Russia	*Ogma decalineatus*	MF683235	USA
*Mesocriconema abolafiai* n. sp.	MN334228	Iran	*Paratylenchus hamatus*	KF242257	USA
*M. curvatum*	HM116062	USA	*Tylenchulus semipenetrans*	JN112274	USA
*M. curvatum*	HM116064	USA	*T. semipenetrans*	FJ588909	China
*M. curvatum*	HM116066	USA	*T. semipenetrans*	MH124562	China
*M. curvatum*	HM116067	USA	*T. semipenetrans*	MH124561	China
*M. curvatum*	HM116068	USA			

#### Remark

The genus *Mesocriconema* has a large number of species, which are morphologically very close to each other. Powers et al. (2016) distinguished 24 COI haplotype groups; only five of them corresponded to morphologically characterized species. The authors further noticed that it is not unexpected that additional sampling of *Mesocriconema* will continue to reveal cryptic species within Linnaean morphospecies; as these species have been described in the recent works (Powers et al., 2016; Olson et al., 2017).

*Mesocriconema abolafiai* n. sp. comes close to *M. ozarkiense* and some other species bearing conical tails with narrow tails (Table 5); however, detailed morphological observations made by light microscopy and SEM, as well as molecular phylogenetic analysis using different genes allowed us to consider *M*. *abolafiai* n. sp. as a new species. The new species can be differentiated from the most closely related species, *M. ozarkiense*, by some morphological and morphometric characters, as well as a different phylogenetic position in the ITS rRNA tree which resulted from a 129 bp (32%) nucleotide divergence. Unfortunately, there is no molecular information on 28S rRNA and 18S rRNA genes of *M. ozarkiense* so the above results can be further supported by additional analyses of these gene sequences.

**Table 5.** Summary of the diagnostic characteristics of 12 species of *Mesocriconema* recently described worldwide. For other species, see (Brzeski et al., 2002). (St measurements in μm, L measurements in mm).

Species	St	R	Rex	RV	RVan	Ran	V	VL/VB	Anas.	A. M.	Vagina	A. V. lip	Tail shape	L	S. M. L.	Original des.
*M. abolafiai* n. sp.	52–60	90–113	19–30	11–14	3–5	7–10	87–90	1.5–1.8	1	smo.–irr.	straight	simple	con–acute	0.4–0.61	flat	Present study
*M. apurense*	48–52	140–147	36–38	9–12	1–2	8–9	93–95	–	0	smo.	straight	lobulated, without projections	con.–rounded	0.38–0.45	rounded	Crozzoli and Lamberti (2001)
*M. campbelli*	58–62	102–108	25–28	11–15	–	5–7	89–93	1.2–1.8	rare	smo.–ser.	straight	serrated	con.–pointed	0.37–0.45	–	Wouts (2006)
*M. juliae*	79–86	89–95	26	8–9	1–2	5–6	91–92	–	few	smo.	straight	with two projections	conical	0.46–0.51	flat	Crozzoli and Lamberti (2001)
*M. lamothei*	65–70	89–98	17–31	6–8	0–2	4–8	91–95		rare	smo.	slightly curved	with two projections	con.–truncate	0.40–0.49	rounded	Cid del Prado Vera (2009)
*M. lobellum*	51–60	85–92	25	6–7	2–3	3–4	92–93	0.9–1.2	rare	–	straight	simple	rounded	0.41–0.53	rounded	Pramodini et al. (2007)
*M. malagutii*	45–49	108–114	33–35	8–9	2	4–6	93–94	–	many	smo.	straight	lobulated, without projections	con.–truncate	0.34–0.40	rounded	Crozzoli and Lamberti (2001)
*M. nebraskense*	45–59	84–113	24–31	6–11	2–3	3–7	90–96	0.7–1.6	1–4	smo.	straight	with two projections	rounded	0.39–0.60	rounded	Olson et al. (2017)
*M. ovospermatum*	82	109	38	9	3	6	93	–	0	finely crenate	straight	?	conical	0.22	?	Mohilal and Dhanachand (1998)
*M. ozarkiense*	49–61	107–119	27–34	10–14	2–4	6–10	89–93	1.0–1.4	0–1	som.–irr.	straight	simple	conical	0.38–0.51	flat	Cordero et al. (2012)
*M. theobromae*	47–51	73–74	24–26	7	1	5	93–94	–	many	som.	straight	lobulated, without projections	conical	0.27–0.32	rounded	Crozzoli and Lamberti (2001)
*M. waitha*	66–78	117-140	26–31	9–10	4–5	4–6	92–94	0.9–1.0	many	som.-irr.	sigmoid	simple	rounded	0.43-0.53	flat?	Pramodini et al. (2006)

**Notes:** St, stylet; Anas., Anastomoses; A. M., Annuli margin; A. V. lip, Anterior vulval lip; S. M. L, Submedian lobes; smo., smooth; ser., serrate; irr., irregular; con, conical; Original des., Original description. **Note1**: *Mesocriconema bakeri* (Wu, 1965; Loof and De Grisse, 1989); *M. calvatum* (Eroshenko, 1982; Loof and De Grisse, 1989); *M. hymenophorum* (Wouts and Sturhan, 1999); *M. longistyletum* (De Grisse and Maas, 1970; Loof and De Grisse, 1989); *M. paramonovi* (Razzhivin, 1974; Loof and De Grisse, 1989); *M. variabile* (Raski and Golden, 1966; Brzeski et al., 2002) and *M. yukonense* (Ebsary, 1982, 1991) that have been listed in Brzeski et al. (2002), transferred to *Neobakernema* (Ebsary, 1981b) by Geraert (2010). **Note2**: *Mesocriconema incrassatum* (Raski and Golden, 1966; Loof and De Grisse, 1989) that has been listed in Brzeski et al. (2002), transferred to *Lobocriconema* (De Grisse and Loof, 1965) by Geraert (2010).
